# Conflicting selection alters the trajectory of molecular evolution in a tripartite bacteria–plasmid–phage interaction

**DOI:** 10.1111/mec.14080

**Published:** 2017-04-03

**Authors:** Ellie Harrison, James P. J. Hall, Steve Paterson, Andrew J. Spiers, Michael A. Brockhurst

**Affiliations:** ^1^ Department of Animal and Plant Sciences University of Sheffield Sheffield S10 2TN UK; ^2^ Institute of Integrative Biology University of Liverpool Liverpool L69 7ZB UK; ^3^ SIMBIOSIS Centre University of Abertay Dundee DD1 1HG UK

**Keywords:** bacteria, coevolution, experimental evolution, microbial biology, molecular evolution, species interactions

## Abstract

Bacteria engage in a complex network of ecological interactions, which includes mobile genetic elements (MGEs) such as phages and plasmids. These elements play a key role in microbial communities as vectors of horizontal gene transfer but can also be important sources of selection for their bacterial hosts. In natural communities, bacteria are likely to encounter multiple MGEs simultaneously and conflicting selection among MGEs could alter the bacterial evolutionary response to each MGE. Here, we test the effect of interactions with multiple MGEs on bacterial molecular evolution in the tripartite interaction between the bacterium, *Pseudomonas fluorescens*, the lytic bacteriophage, SBW25φ2, and conjugative plasmid, pQBR103, using genome sequencing of experimentally evolved bacteria. We show that individually, both plasmids and phages impose selection leading to bacterial evolutionary responses that are distinct from bacterial populations evolving without MGEs, but that together, plasmids and phages impose conflicting selection on bacteria, constraining the evolutionary responses observed in pairwise interactions. Our findings highlight the likely difficulties of predicting evolutionary responses to multiple selective pressures from the observed evolutionary responses to each selective pressure alone. Understanding evolution in complex microbial communities comprising many species and MGEs will require that we go beyond studies of pairwise interactions.

## Introduction

Bacteria engage in complex networks of ecological interactions both with other species and with a wide diversity of mobile genetic elements (MGEs), including phages, plasmids and transposons. These MGEs play a central role in bacterial evolution as vectors of horizontal gene transfer (Jain *et al*. 2003), but also act as important sources of selection on bacterial populations. Pairwise coculture studies have demonstrated that both phages and plasmids can be important drivers of bacterial evolution. Phages are a major cause of bacterial mortality (Bouvier & del Giorgio 2007), and antagonistic co‐evolution between bacteria and phage generates strong, reciprocal selection for adaptations in phage infectivity and bacterial resistance traits (Buckling & Rainey 2002) resulting in rapid genomic evolution of both phage (Paterson *et al*. 2010) and bacteria (Scanlan *et al*. 2015). Plasmids, meanwhile, often carry large cargos of accessory genes as well as bacterial regulatory elements (Tett *et al*. 2007; Smillie *et al*. 2010), and so can place a considerable fitness cost on the host cell. The additional genetic material can impose a physiological burden by expressing and translating plasmid genes, whereas regulatory elements can cause disruption of bacterial intracellular regulatory networks (Baltrus 2013). Coculture experiments suggest that plasmid carriage selects for compensatory mutations, either on the chromosome or on the plasmid, which ameliorate the cost of plasmid carriage (Harrison *et al*. 2015a; San Millan *et al*. 2015; Yano *et al*. 2016). For the majority of bacteria–plasmid interactions, because rates of conjugative transfer are typically too low to counteract purifying selection against costly plasmids (Levin 1993; Bergstrom *et al*. 2000), compensatory evolution is thought to be important for the survival of plasmids in bacterial populations (Harrison & Brockhurst 2012). While pairwise coculture experiments reveal the impact of MGE‐mediated selection on bacterial evolution, studies to date typically consider the effect of each MGE in isolation. However, in nature bacteria are likely to interact with multiple MGEs simultaneously and therefore will experience concurrent selective pressures. An important question is to understand how these constituent pairwise interactions between bacterial hosts and MGEs combine in more complex communities to determine the overall effect on bacterial genomic evolution.

The effect of multiple, concurrent selective pressures on bacterial evolution will depend upon the relative strengths of selection and the genetic correlations between the traits under selection. An overview of these potential effects is given in Table S1 (Supporting information). In general, it is likely that stronger selective pressures will outweigh weaker ones due to clonal interference (Gerrish & Lenski 1998; Rozen *et al*. 2002); for example, selection arising from antagonistic co‐evolution weakens the evolutionary response to abiotic selection in phages (Zhang & Buckling 2011). Furthermore, changes to host demography can alter the strength of selection from interacting partners. For instance, reductions in bacterial density due to protist predation lead to reduced encounter rates between bacteria and phages, and consequently weaker bacterial evolutionary responses to phage attack (Friman & Buckling 2013). Genetic correlations between the traits under selection can range from positive to negative and can arise via a range of mechanisms including pleiotropy (Bohannan & Lenski 2000; Örmälä‐Odegrip *et al*. 2015), epistasis (Buckling *et al*. 2006; Scanlan *et al*. 2015) or linkage. Positive genetic correlations, for example where a mutation conferring resistance against one enemy pleiotropically enhances resistance to a second enemy, favour the evolution of generalist prey defences in multi‐enemy communities (Örmälä‐Odegrip *et al*. 2015). By contrast, negative genetic correlations are likely to constrain evolution to one or both selective pressures. For example, negative epistasis between mutations conferring phage resistance and mutations conferring environmental adaptation constrained bacterial abiotic adaptation in populations co‐evolving with phages (Scanlan *et al*. 2015). However, because relatively few studies have identified the causative mutations, we have only a limited understanding of how multiple selective pressures shape bacterial molecular evolution.

In this study, we used genome sequencing to contrast the evolutionary responses of bacteria to abiotic selection, phage selection, plasmid selection and combined phage–plasmid selection. Our experimental system comprised a conjugative mercury resistance plasmid, pQBR103; a lytic phage, SBW25φ2; and their shared bacterial host, *Pseudomonas fluorescens* SBW25. In a previous study, we experimentally evolved *P. fluorescens* populations under four treatments – MGE‐free, phage‐only, plasmid‐only or phage–plasmid together – and reported the effects of plasmid carriage on bacteria–phage co‐evolution at the phenotypic level. In populations co‐evolving with phages, plasmids constrained bacterial evolution of resistance to phage infection relative to plasmid‐free bacteria. This reduction in the strength of phage resistance was unlikely to have been driven by demographic effects as plasmid carriage was not associated with reduced bacterial or phage population density. However, plasmids did select for the evolution of higher frequencies of mucoidy in bacterial populations in response to phage attack. Mucoidy is caused by overproduction of exopolysaccharides (Hay *et al*. 2014) and confers partial resistance to phage infection (Scanlan & Buckling 2012). Thus, plasmids altered the phenotypic evolutionary responses of bacteria to phage selection. Here, we additionally characterize the effect that phage selection had on bacterial evolutionary responses to plasmid selection, in particular compensatory evolution. We determine how bacterial molecular evolution varied between the four treatments allowing us to compare evolution between bacterial hosts and MGEs under pairwise vs. tripartite interactions.

## Methods

For this study, we sequenced single bacterial clones from the experiment described in Harrison *et al*. (2015b). In brief, six replicate populations of the bacteria *P. fluorescens* SBW25, each initiated from a single bacterial colony, were allowed to evolve either with or without the mercury resistance plasmid pQBR103 (Tett *et al*. 2007), and in the presence and absence of the lytic phage SBW25φ2 (Buckling & Rainey 2002). Populations were grown in King's B (KB) with shaking at 28 °C and propagated by 1% serial transfer for 20 transfers corresponding to approximately 150 bacterial generations. Populations were evolved in sub‐MIC levels of Hg(II) (8 μm HgCl_2_) (Harrison *et al*. 2015b). These conditions provided sufficient positive selection on plasmid‐borne mercury resistance genes to ensure plasmid maintenance but are permissible for plasmid‐free bacteria that do not carry the *mer* operon (Harrison *et al*. 2015b). For this study, we randomly selected a single clone, representative of the majority phenotype, from each of the 24 populations from the final time point. Clones were sequenced on the Illumina MiSeq platform and reads aligned using BWA. Small variants (SNPs and small indels) were identified using GATK HaplotypeCaller and SNPeff and structural rearrangements identified using BreakDancer. A list of all mutational targets is given in Table S2 (Supporting information).

To investigate the dynamics of compensatory mutations associated with plasmid amelioration, we screened 20 clones per population at four transfer intervals (transfers 0, 4, 8, 12, 16 and 20) for exoprotease production. Both this and previous experiments have revealed extensive parallel evolution of the bacterial GacAS system, which controls a large suite of secreted proteins, including exoproteases (Cheng *et al*. 2013). GacAS function was detected by spotting ~1 μL of overnight culture onto skimmed milk agar (10% milk powder in nutrient broth agar) alongside GacAS positive and negative controls (Harrison *et al*. 2015a). Colonies were grown at 28 °C for 24 h and then at room temperature for 24 h. Clones able to produce exoproteases can be identified by a halo around the colony, due to digestion of casein in the media (Cheng *et al*. 2013).

## Results

### Rates of bacterial genome evolution

Evolved clones contained between one and six mutations, the vast majority of which were predicted to have strong phenotypic effects, with only two synonymous mutations identified among the 24 sequenced clones. Average pairwise genetic distance from the ancestor varied between treatments (anova_effect of treatment_: *F*_3,20_ = 5.49, *P* = 0.0064); this effect was largely driven by phage selection accelerating bacterial evolution relative to the MGE‐free controls (average no. of mutations under ‘phage‐only’ 4 ± 0.58SE vs. ‘MGE‐free’ 1.17 ± 0.17SE: post hoc comparison, *t *=* *3.97, *P* = 0.0039). In contrast, phage selection did not lead to accelerated evolution in plasmid‐carrying bacteria (no. of mutations under ‘phage–plasmid’ 2.17 ± 0.60SE vs. ‘MGE‐free’: post hoc comparison, *t *=* *1.403, *P* = 0.512). Thus, the plasmid appears to have constrained the bacterial response to phage selection.

### Targets of selection varied between treatments

**Figure 1:**
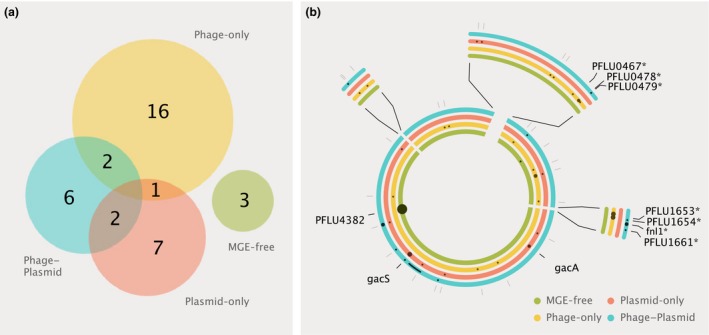
Bacterial genome evolution in the presence and absence of symbionts. (a) Venn diagram comparing loci targetted across six replicate clones shared within and between each treatment. (b) Summary of mutations identified across six sequenced clones for each treatment. Circles represent a summarized bacterial genome for each MGE treatment, with dots representing loci targetted by mutations across the six replicate clones per treatment. Dots are scaled by the number of times mutations appear across replicates. Expanded sections are shown for clarity. Loci highlighted in the text are named, with LPS‐associated loci indicted by *.

Loci targeted by mutations varied significantly between treatments. Bacterial clones were more likely to share mutational targets with those from replicate populations from the same treatment than with those from different treatments [permutational manova (Scanlan *et al*. 2015), *N* = 1000, *F* = 3.05, *P* = 0.001]. Notably, clones that had evolved without MGEs shared no mutational targets with clones from the MGE treatments (Fig. 1a). Mutations identified in the six clones from the MGE‐free treatment affected just two loci: PFLU0956, which was mutated in 1/6 clones from this treatment and is known to be associated with laboratory adaptation (Lind *et al*. 2015); and PFLU4382, which was mutated in all six clones (one clone had a mutation affecting the promotor region, while all others had mutations disrupting the coding sequence). PFLU4382 is predicted to be a thiol–disulphide interchange protein associated with protein folding (Silby *et al*. 2009). Mutations at this locus are likely to be involved in ameliorating mercury toxicity, as mercury binds to thiol groups and interferes with the formation of disulphide bonds (Sharma *et al*. 2008). Given this role, it is perhaps unsurprising that these mutations were not observed in clones carrying the plasmid which can efficiently detoxify mercury via the mercury resistance operon (*mer*). However, it is notable that in the phage‐only treatment, where increased mercury tolerance would likely have been beneficial, PFLU4382 evolution was not observed, suggesting that phage selection impeded environmental adaptation, as has been observed previously (Scanlan *et al*. 2015).

### Plasmid carriage alters the rate and trajectory of the bacterial evolutionary response to phages

Mutational targets were more variable among bacterial clones from the MGE treatments. The majority of clones (13/18), in particular in the phage‐only treatment (6/6), carried at least one singleton mutation (i.e. mutational targets unique to one clone). However, overall, mutations were not randomly distributed, as even among the MGE treatments mutational targets were still more likely to be shared within than between treatments (Fig. 1a; permutational manova excluding the MGE‐free treatment, *N* = 1000, *F* = 1.69, *P* = 0.026).

Excluding genes of unknown function, a large proportion of the mutational targets in the phage‐only and phage–plasmid selection treatments were in lipopolysaccharide (LPS) biosynthesis‐associated loci, which have been linked to evolved phage resistance (Scanlan *et al*. 2015). By contrast, no LPS mutations were identified in clones evolved without phage selection. Consistent with the reduced rate of phage resistance evolution observed in plasmid‐carrying bacterial populations (Harrison *et al*. 2015b), we observed a higher frequency of mutations in LPS‐associated loci in clones from the phage‐only treatment (on average 1.76 mutations/clone) compared to those from the phage–plasmid treatment (on average 0.83 mutations/clone). Moreover, the targets of selection differed somewhat between the phage‐only and the phage–plasmid treatments (Fig. 1b). In total, seven LPS‐associated loci were targeted in the 12 clones co‐evolved with phages. Five of these evolved in parallel (i.e. in >1 clone), and of these loci, three were treatment‐specific. Two loci (PFLU0478 and PFLU1653) were unique to the phage‐only treatment, each targeted in two clones, and have been previously associated with evolved phage resistance in SBW25 (Scanlan *et al*. 2015). One locus, *fnl1*, was unique to the phage–plasmid treatment and was found in two clones, both of which had evolved the mucoid colony phenotype. *fnl1* is a homolog of *fnlA*, which has been associated with capsule production in *Pseudomonas aeruginosa* and *Staphylococcus aureus* (Mulrooney *et al*. 2005). Although the genetics which underlie the mucoid phenotype are not well understood (Scanlan & Buckling 2012), it is likely that the mutations in *fnl1* contribute to mucoidy as the phenotype is caused by overproduction of alginate, a polysaccharide involved in bacterial capsule formation.

### Phage selection impedes compensatory evolution in plasmid‐carriers

Comparison of the plasmid‐only and phage–plasmid treatments reveals a clear difference in the loci targeted by mutations between these treatments. Notably, 5/6 bacterial clones from the plasmid‐only treatment carried mutations in the *gacA/gacS* two‐component global regulatory system, whereas mutations in these loci were observed in only 1/6 clones from the phage–plasmid treatment (Fig. 1b; clone‐level mutations shown in Fig. S1, Supporting information). The lower frequency of mutations in *gacA/gacS* under phage selection suggests that co‐evolving with phages constrained the potential for bacteria to ameliorate the cost of plasmid carriage. Loss‐of‐function mutations in *gacA* or *gacS* have previously been shown to be an important mechanism of compensatory evolution in this bacteria–plasmid association, occurring with a high degree of parallelism across varied selective environments and completely ameliorating the cost of plasmid carriage (Harrison *et al*. 2015a). It is possible that compensatory evolution may have been achieved through alternative mechanisms in the presence of phages. Five of six sequenced clones from the phage–plasmid treatment contained mutations whose functional effects are currently unclear. However, it should be noted that unlike the highly parallel evolution of *gacA* and *gacS* mutations across evolutionary replicates, the majority of other mutations observed in the phage–plasmid treatment occurred in only a single strain suggesting that they are unlikely to be under strong selection.

**Figure 2:**
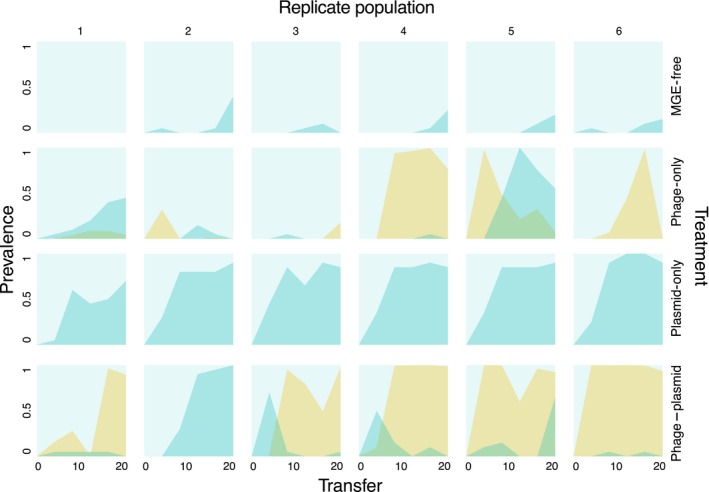
The population dynamics of mucoid and GacAS‐deficient genotypes in replicate populations. Twenty‐four colonies collected from evolving populations at four transfer intervals were screened for exoprotease production, a phenotype associated with GacAS function. The proportion of exoprotease‐negative mutants is shown for each replicate population through time (blue), overlaid with the proportion of mucoid mutants (yellow) in the same population (data from Harrison *et al*. 2015b).

To further understand the dynamics of *gacA/gacS* compensatory mutations, we tracked the frequency dynamics of exoprotease production, which is positively regulated by GacAS and therefore an indicator of GacAS function (Cheng *et al*. 2013) (Fig. 2). Loss of exoprotease production occurred early and swept to high frequency in all six populations of the plasmid‐only selection treatment, including the population where the sequenced clone did not contain a *gacA* or *gacS* mutation. In agreement with the sequencing, this particular clone was positive for exoprotease production but was in the minority (2/20 clones tested) within the population. By contrast under phage–plasmid selection, loss of exoprotease function, although detectable, only reached high frequency in 1/6 populations, consistent with the sequencing data. Intriguingly, this population was also distinct from the other replicates within the phage–plasmid treatment in that it was the only population in which the mucoid colony morphology did not sweep to high frequency (Fig. 2); moreover, it displayed a high level of complete resistance against its phage population (Fig. S2, Supporting information) (Harrison *et al*. 2015b). Notably, the clone sequenced from this population carried a mutation in LPS‐associated locus PFLU0479 which was also targeted by a clone from the phage‐only treatment. This suggests a potential link between GacAS function and the evolution of mucoid‐associated phage resistance.

## Discussion

We show that individually plasmids and phages impose selection leading to bacterial evolutionary responses that are distinct from bacterial populations evolving without MGEs, but that together plasmids and phages impose conflicting selection on bacteria, constraining the evolutionary responses observed in pairwise interactions. Without MGE selection, bacteria adapted to the low concentration of Hg(II) present in the growth medium via parallel mutations to PFLU4382, a thiol–disulphide interchange protein involved in protein folding. Phage‐only selection led to high rates of bacterial evolution, in particular at LPS‐associated loci linked to phage resistance (Scanlan *et al*. 2015), but bacterial evolution in response to phages was constrained in the presence of plasmids and in some cases targeted different genes. Plasmid‐only selection led to the evolution of parallel compensatory mutations at *gacA/gacS*, which have previously been shown to completely ameliorate the cost of plasmid carriage (Harrison *et al*. 2015a), an evolutionary trajectory that was impeded when co‐evolving with phages in the phage–plasmid treatment.

Constraints on evolutionary responses to conflicting selective pressures are predicted where there are negative genetic correlations between the traits under selection. This mechanism may explain the lower frequency of *gacA/gacS* compensatory mutations observed in the phage–plasmid treatment compared to the plasmid‐only treatment. GacAS is a global regulator that positively controls biosynthesis of a large suite of secreted molecules (Cheng *et al*. 2013), including capsular polysaccharides such as alginate (Cheng *et al*. 2013; Hay *et al*. 2014), the overproduction of which causes mucoidy (Hay *et al*. 2014). Loss‐of‐function mutations in *gacA* or *gacS* to ameliorate the cost of plasmid carriage would have likely prevented the evolution of the mucoid phenotype, the form of partial phage resistance which evolved to high frequency in the phage–plasmid treatment (Harrison *et al*. 2015b). Phage‐imposed selection for mucoidy could therefore have prevented compensatory evolution through loss of GacAS function. This inference is further supported by the genetic and phenotypic data from the single replicate population from the phage–plasmid treatment that did not evolve the mucoid phenotype: here, loss of GacAS function swept to high frequency, suggesting that this evolutionary trajectory is mutually exclusive from the evolution of mucoidy. Compensatory evolution is thought to be a key factor stabilizing plasmids in bacterial populations, and we have previously demonstrated that mutations in *gacA* or *gacS* are extremely efficient at ameliorating the cost of pQBR103 in this bacterial host (Harrison *et al*. 2015a). By impeding this important route to compensatory evolution, phage selection could limit the conditions allowing the survival of plasmids, and in so doing restrict the potential for future horizontal gene transfer within the bacterial community.

Nevertheless, the observed evolution of high frequencies of mucoidy in the phage–plasmid treatment is somewhat counterintuitive, because it not only limits the available mechanisms for compensatory evolution but also confers weak partial resistance to phage infection when other forms of complete resistance via modification of LPS phage binding sites were readily available. Why, then, was mucoidy favoured in the presence of plasmids? A possible explanation is that acquisition of large plasmids, such as pQBR103, can have major effects on the bacterial phenotype, beyond those of the accessory gene cargo itself, through large‐scale remodelling of bacterial gene regulatory networks (Dougherty *et al*. 2014). We have previously shown that acquisition of the pQBR103 plasmid causes ~17% of SBW25 chromosomal genes to be upregulated, including a large proportion of the alginate biosynthesis pathway (seven positive regulators, three negative regulators and the biosynthesis locus PFLU5986; Harrison *et al*. 2015a). Thus, plasmid‐mediated alterations to bacterial gene expression could have primed plasmid‐carrying cells for the evolution of mucoid‐based resistance against phages. Subsequent mutations, for example those occurring in the *fnl1* loci, may have led to genetic assimilation (Pigliucci *et al*. 2006) of these regulatory effects of plasmid carriage. Moreover, the emergence of mucoid‐based resistance in the phage–plasmid treatment may in turn have weakened selection for mutations conferring complete resistance. Thus, plasmid‐mediated alterations to the intracellular environment of bacteria, especially through regulatory disruption, could have important evolutionary consequences, altering the trajectory of subsequent bacterial evolution.

These data show that multiple MGEs can impose conflicting selective pressure on bacterial populations, leading to divergent outcomes for bacterial molecular evolution. Differences in evolutionary trajectory can have important ecological implications in microbial communities (McClean *et al*. 2015; Beaume *et al*. 2017). The evolution of the mucoid phenotype under coselection by phage and plasmids observed here is likely to alter the local environment, increasing viscosity and leading to formation of biofilms that are resilient to environmental stressors (Hentzer *et al*. 2001). In addition, mucoidy has also been associated with reduced receptiveness to plasmid conjugation (Pérez‐Mendoza & de la Cruz 2009) potentially limiting rates of horizontal gene transfer. Moreover, by limiting the bacterial response to phage‐mediated selection, plasmids could stabilize bacteria–phage coexistence (Wright *et al*. 2016) which can be destabilized due to asymmetries in the evolutionary potential for phage infectivity compared to bacterial resistance (Buckling & Brockhurst 2012). However, given the role of phages as drivers of bacterial diversity (Koskella & Brockhurst 2014), changes to, and perhaps weakening of, these co‐evolutionary interactions are likely to have important implications for natural microbial communities. Intriguingly, conflicting selection appears to arise due to the negative pleiotropic effects of the mutations that compensate for the cost of plasmid carriage: whereas loss of the global regulator GacAS allows bacteria to ameliorate the cost of plasmid carriage, it concomitantly prevents them from evolving mucoid‐based resistance against phage infection. While the molecular details underpinning genetic correlations will vary between systems, these findings highlight the likely difficulties of predicting evolutionary responses to multiple selective pressures from the evolutionary responses observed to each selective pressure alone. Understanding evolution in complex microbial communities comprising many species and MGEs will require that we go beyond studies of pairwise interactions.

## Conflict of interest

The authors declare that this research was conducted in the absence of any commercial or financial relationships that may cause a potential conflict of interest.

## Data accessibility

Sequencing data are available via the European Nucleotide Archive (PRJEB19606), and phenotypic data and variant call data are available on Dryad (doi:10.5061/dryad.3hk0v).

M.A.B., S.P. and A.J.S. obtained the funding. E.H., M.A.B devised the project. E.H., J.H. and M.A.B. wrote the manuscript.

## Supporting information


**Fig. S1** Mutations identified in evolved clones.
**Fig. S2** Bacterial sensitivity to phage infection.
**Table S1** The potential effects of community interactions on evolution of a focal species.
**Table S2** Genomic changes in response to evolution.
