# Supplementary material for: Conflicting selection alters the trajectory of molecular evolution in a tripartite bacteria–plasmid–phage interaction
Source: Mol Ecol. 2017 Apr 3;26(10):2757–64. doi: 10.1111/mec.14080 (PMC5655702; doi:10.1111/mec.14080)
Supplement: Supplementary file 1 — Fig. S1 Mutations identified in evolved clones. Fig. S2 Bacterial sensitivity to phage infection. Table S1 The potential effects of community interactions on evolution of a focal species. Table S2 Genomic changes in response to evolution. [file MEC-26-2757-s001.pdf]

SUPPORTING INFORMATION:

Conflicting selection alters the trajectory of molecular evolution in a tripartite bacteria-plasmid-phage interaction

Ellie Harrison[1], James J. P. Hall[1], Steve Paterson[2], Andrew J. Spiers[3] & Michael A. Brockhurst[1]

[1]Department of Animal and Plant Sciences, University of Sheffield, Sheffield, S10 2TN, Sheffield, S10 2TN, UK. [2]Institute of Integrative Biology, University of Liverpool, Liverpool, L69 7ZB, UK [3]SIMBIOSIS Centre, University of Abertay, Dundee, DD1 1HG, UK

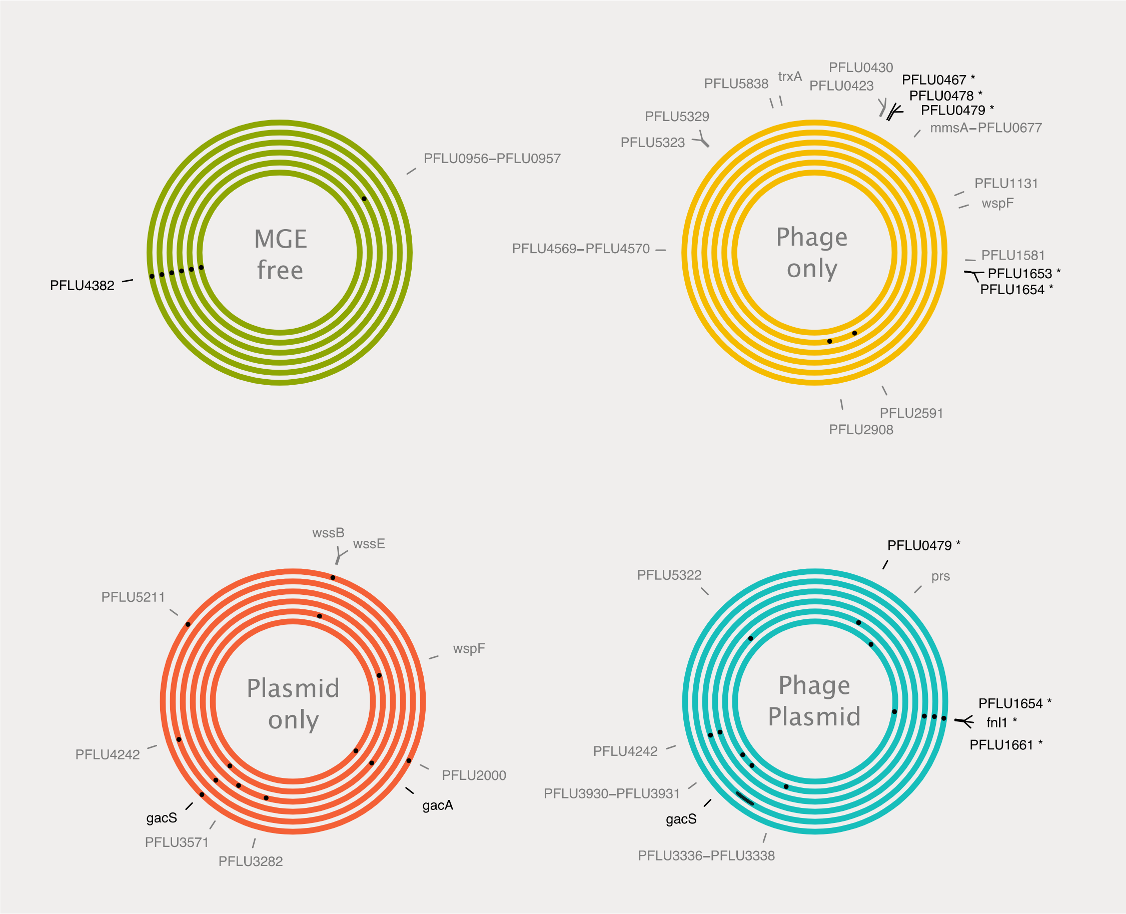

Fig. S1. Mutations identified in evolved clones. Circles represent the bacterial genomes of each sequenced clone taken from the 4 MGE treatments. Dots represent loci targetted by mutations. Loci high lighted in the text are shown in black, with LPS-associated loci indicted by *.

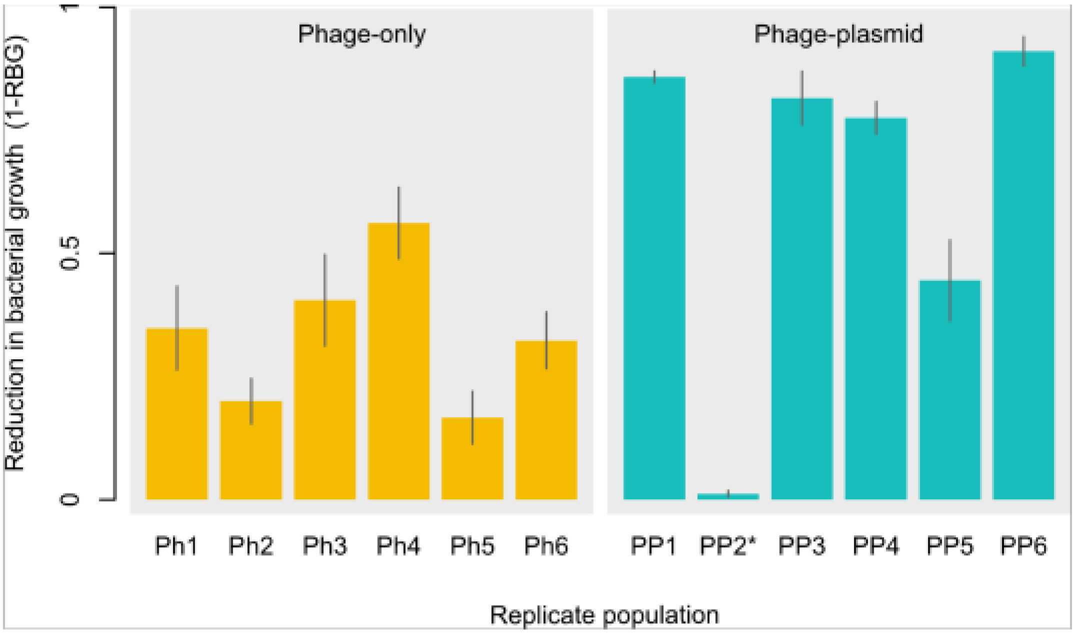

Fig. S2. Bacterial sensitivity to phage infection. Reduction in Bacterial Growth due to phage infection (1-RBG) was estimated for 20 clones per populations by comparing bacterial growth in the presence and absence of their allopatric phage population. Lines show standard error of population means (n=6). Data taken from Harrison et al. (2015b). The single phage-plasmid population which displayed high rates of mucoidy by low rates of gacA/gacS compensatory mutation is indicated by *.

EFFECT	OUTCOME
Demographic	
**Population density** – Interactions with other species may reduce (e.g. predation, parasitism, competition) or increase (e.g. mutualism) population density of one or both species and can alter the strength of interactions with other connect species.	
e.g. Reductions in population density in one species due to predation from A may lead to a lower encounter rate with predator B.	Reduced strength of selection from B
**Clonal interference** – The spread of beneficial mutations on separate genetic backgrounds can prevent one or both from reaching fixation. In communities, mutations that are favoured under selection from one interaction partner may hinder the spread of mutations favoured under another.	Reduced rate of evolution, particularly to weaker sources of selection.
Genetic correlations	
**Negative pleiotropy** – Mutations favoured in the presence of one interaction are costly in the presence of another interaction e.g. where increased resistance to one predator increases susceptibility to another.	The presence of the second partner **reduces** the net benefit of mutations that are otherwise beneficial.
**Positive pleiotropy** – Mutations which are beneficial in the presence of one interaction are also beneficial in the presence of a second interaction, e.g. mutations which confer weak resistance to one partner may also confer resistance to another.	The presence of the second partner **increases** the net benefit of mutations. Mutations that confer weaker but generalist fitness effects are more likely to spread.
**Negative Epistasis** – The phenotype (here we are generally referring to fitness) of an individual carrying 2 mutations is reduced compared with the additive effects of each mutation alone.	Mutations that may be beneficial in the context of one interaction are **less beneficial/costly** in genetic backgrounds containing mutations favoured in the presence of another interaction.
**Positive Epistasis** – The phenotype of an individual carrying 2 mutations is greater that would be expected from each mutation alone.	Mutations that may be beneficial in the context of one interaction are **more beneficial** in genetic backgrounds containing mutations favoured in the presence of another interaction.
**Linkage disequillibrium** – The co-inheritance of alleles. As bacteria are asexual linkage disequilibrium tends to be strong, meaning that the genetic correlations are less likely to break down.	The spread of mutations favoured under one interaction will be constrained where they are co-inherited with alleles that are costly in the presence of another (e.g through negative epistasis)

Table S1 | The potential effects of community interactions on evolution of a focal species.

Functional group	Gene name / locus ID	Product	Clone ID			
**Cellulose**	***wspF* / PFLU1224**	**Not assigned**		P2 ••	Ph2 • Ph5 •	
	wssB / PFLU0301	Not assigned		P2 •		
	wssE / PFLU0304	Not assigned		P6 ••		
**Energy**	*mmsA*-PFLU0677	Not assigned - putative paraquat-inducible protein			Ph2 —	
	PFLU0423	putative fatty acid biosynthesis-related protein			Ph6 ••	
**GacA/S**	**gacA / PFLU2181**	**Response regulator in two component regulatory system**		P1 • P3 •		
	**gacS / PFLU3777**	**histidine kinase in two component regulatory system**		P6 • P4 • P2 •		PP2 •
**LPS**	PFLU0467	putative lipopolysaccharide biosynthesis-related protein			Ph2 ••	
	**PFLU0478**	**putative glycosyl transferase**			Ph1 • Ph5 ••	
	**PFLU0479**	**putative glycosyl transferase**			Ph2 •	PP2 •
	**PFLU1653**	**putative transport-related 2C membrane protein**			Ph6 • Ph4 •	
	PFLU1652-PFLU1653	putative membrane protein - putative transport-related 2C membrane protein			Ph3 —	
	**PFLU1654**	**conserved hypothetical protein**			Ph4 •• Ph3 •• Ph6 ••	PP5 •
	***fnl1* / PFLU1657**	**polysaccharide biosynthesis protein**				PP6 •• PP1 •
	PFLU1661	putative pilin glycosylation protein				PP4 •
**Membrane**	PFLU1131	putative two-component system 2C sensor kinase			Ph5 •	
	PFLU1581	putative exported protein			Ph6 ••	
	PFLU5211	putative membrane protein		P6 •		
	PFLU2000	putative membrane protein		P6 •		
**Missfolded protein repair**	PFLU4381-PFLU4382	two-component system response regulator - thiol:disulfide interchange protein	F2 —			
	**PFLU4382**	**thiol:disulfide interchange protein**	F3 •			

			F1 • F4 • F5 •• F6 •			
**Nucleotide biosynthesis**	*prs* / PFLU0732	ribose-phosphate pyrophosphokinase				PP1 •
**Ribosome binding**	PFLU3930-PFLU3931	trigger factor - FolD bifunctional				PP2 —
**Signal transduction mechanisms**	PFLU5329	putative putative sensory box GGDEF/EAL domain protein			Ph1 •	
**unknown**	PFLU0430	putative histidine ammonia-lyase			Ph5 ••	
	PFLU0956-PFLU0957	putaive GGDEF domain membrane protein - putative exported protein	F3 —			
	PFLU2591	conserved hypothetical protein			Ph2 •	
	PFLU2908	hypothetical protein			Ph2 •	
	PFLU3282	putative short chain dehydrogenase		P3		
	PFLU3336-PFLU3338	short chain dehydrogenase - probable zinc-binding dehydrogenase				PP2 —
	PFLU3571	putative GGDEF domain signaling protein		P3 •		
	**PFLU4242**	**conserved hypothetical protein**		P5 •		PP3 • PP4 •
	PFLU4569-PFLU4570	conserved hypothetical protein - fumarate and nitrate reduction regulatory protein			Ph1 —	
	PFLU5322	conserved hypothetical protein				PP2 •
	PFLU5323	putative exported protein			Ph5 •	
	PFLU5838	putative penicillin amidase			Ph4	
	trxA / PFLU5901	Not assigned			Ph4 •	
	171Kb duplication					PP5 ••

Table S2 | Genomic changes in response to evolution. Loci and intergenic regions (surrounding loci shown) targeted by mutations among sequenced clones from replicate populations. Loci and are grouped by function and dashed lines indicate operon groupings. Loci which have been targeted independently in multiple clones are highlighted in bold. Clones IDs are derived from the treatment; F – MGE-free, P – plasmid only, Ph – Phage only, PP plasmid-phage, and the replicate population number; 1-6. Symbols indicate the severity of mutations; • moderate (non-synonymous snps and in frame indels), •• high (nonsense mutations and frame shifts), — intergenic.
